# Seasonal Time Series Forecasting by F^1^-Fuzzy Transform

**DOI:** 10.3390/s19163611

**Published:** 2019-08-19

**Authors:** Ferdinando Di Martino, Salvatore Sessa

**Affiliations:** 1 Dipartimento di Architettura, Università degli Studi di Napoli Federico II, Via Toledo 402, 80134 Napoli, Italy; 2 Centro di Ricerca Interdipartimentale di Ricerca A. Calza Bini, Università degli Studi di Napoli Federico II, Via Toledo 402, 80134 Napoli, Italy

**Keywords:** seasonal time series, F-transform, F^1^-transform forecasting, TSSF, TSSF1

## Abstract

We present a new seasonal forecasting method based on F^1^-transform (fuzzy transform of order 1) applied on weather datasets. The objective of this research is to improve the performances of the fuzzy transform-based prediction method applied to seasonal time series. The time series’ trend is obtained via polynomial fitting: then, the dataset is partitioned in S seasonal subsets and the direct F^1^-transform components for each seasonal subset are calculated as well. The inverse F^1^-transforms are used to predict the value of the weather parameter in the future. We test our method on heat index datasets obtained from daily weather data measured from weather stations of the Campania Region (Italy) during the months of July and August from 2003 to 2017. We compare the results obtained with the statistics Autoregressive Integrated Moving Average (ARIMA), Automatic Design of Artificial Neural Networks (ADANN), and the seasonal F-transform methods, showing that the best results are just given by our approach.

## 1. Introduction

Today, seasonal time series forecasting represents a crucial activity in many fields such as macroeconomics, finance and marketing, and weather and climate analysis. In particular, predicting the evolution of weather parameters as climate change effects represents a crucial activity for the purpose of planning and designing resilient actions to safeguard landscape, biodiversity, and the health of citizens. One of the processes for the evolution of the climate of an area of study is to analyze continuously measured data from weather stations and to capture and monitor changes in seasonal values of climate parameters. In this analysis, a significant role is played by seasonal time series forecasting algorithms applied to weather data. 

Time series forecasting techniques are applied to time-measured data in order to predict future trends of a variable. A characteristic detectable in many time series is seasonality, consisting in a regularly repeating pattern of highs and lows related to specific time periods such as seasons, months, weeks, and so on.

A seasonal behavior is present, generally, in time series of weather variables: it consists of variations that are found with similar intensity in the same periods. For example, the warmest daily temperature is recorded periodically in the summer season.

A cyclical behavior, on the other hand, can drift over time because the time between periods is not precise. For example, the wettest day in a geographical area can often be recorded in autumn, but sometimes, it occurs also in other seasons of the year.

An irregular behavior is observed in time series which present short-term oscillations. Normally, they are caused by a stationary stochastic process.

Many algorithms were proposed in the literature to analyze seasonal and cyclical time series. Treatments of this approaches are in References [1,2,3,4]. The most famous time series forecasting statistical method is the Box–Jenkins approach that applied Autoregressive Integrated Moving Average (ARIMA) models [1,2,3,4]. A specific model, called Seasonal ARIMA or SARIMA [5], is used when the time series exhibits seasonality. 

ARIMA models cannot capture nonlinear tendencies generally present in a time series: some soft computing approaches have been presented in the literature for capturing nonlinear characteristics in seasonal time series.

Artificial Neural Networks (ANN) can be applied as nonlinear auto-regression models to capture nonlinear characteristics in the data. Some authors propose a multilayer Feed Forward Network (FNN) method [6,7] in which the output value *y_t_* of a parameter *y* at time *t* is given by a function of the values *y*_*t*−1_, *y*_*t*−2_, …, *y*_*t*−ND_ of the measured values at time *t* − 1, *t* − 2, …, *t* − ND, where ND is the number of input nodes. Other authors propose seasonal time series forecasting methods based on Time Lagged Neural Networks (TLNN) architecture [8,9,10,11]. In a TLNN, the input nodes are the time series values at some particular lags. For example, in a time series with monthly seasonal periods, the neural network used for forecasting the parameter value at time *t* can contain input nodes corresponding to the lagged values at the time *t* − 1, *t* − 2, ..., *t* − 12.

The main problem of the ANN-based forecasting method is the choice of appropriate values for the network parameters on which the accuracy of the results depends heavily.

Also, Support Vector Machine-based (SVM) approaches are used to capture nonlinear characteristics in time series forecasting. SVM uses a kernel function to transform the input variables into a multidimensional feature space; then, the Lagrange multipliers are used for finding the best hyperplane to model the data in the feature space [12]. Some authors propose seasonal forecasting methods based on Least Squares Support Vector Machine models [13,14,15]. LSSVM [16] is a variation of SVM that involves least square optimization solutions in a kernel-based SVM regression model.

The main advantage of SVM-based methods is that that the solution is unique and there is no risk to move towards local minima, but some problems remain as the choice of the kernel parameters influences the structure of the feature space, affecting the final solution.

In order to overcome these difficulties in Reference [17], a hybrid adaptive ANN method, called ADANN (Automatic Design of Artificial Neural Networks), is proposed by applying a genetic algorithm for evolution of the ANN topology and the back-propagation parameter. The authors compare this algorithm with SARIMA- and SVM-based algorithms on various time series, showing that the best results in terms of accuracy are obtained by using the ADANN algorithm, even if it requires more computational effort than the previous ones.

The Fuzzy Transform (F-transform) technique [18] was applied by some authors in times series forecasting. In Reference [19], the authors use the multidimensional inverse F-transform as a regression function in a time series analysis. In Reference [20], a hybrid method integrating fuzzy transform, pattern recognition, and fuzzy natural logic techniques is proposed in order to predict the trend and the seasonal behavior of seasonal time series.

In References [21,22], a novel forecasting algorithm is proposed by using the direct and inverse F-transform, called the Time Series Seasonal F-transform (TFSS). In the TFSS, a polynomial fitting is applied to evaluate the trend of the time series. Then, the dataset is de-treated by subtracting the trend from it and the de-treated dataset is partitioned in s seasonal subsets. Finally, the inverse F-transform is calculated on each seasonal subset. The authors test the TFSS algorithm on whether the time series shows that it improves the performances of the seasonal ARIMA and F-Transform forecasting methods.

The aim of our research is to improve the performance of the TFSS algorithm. In this work, we apply the inverse F^1^-transform [23] as a regression function to manage seasonal time series: the F^1^-transform represents a refinement of the F-transform for approximating a function. We have implemented a variation of the TFSS method in which we used the F^1^-transform to forecast seasonal time series. We test our method to forecast seasonal time series of the climatic Heat Index (HI) parameter calculated by the daily weather data measured from a set of weather stations. In our experiments, we compare the performances of our method with the ones obtained by using the TSSF, Seasonal Arima, and ADANN methods. In Reference [23], the authors show that SVM and ADANN have the same performances. For this reason, in our experiments carried out in this research, we do not use the SVM method but only the ADANN method

In Section 2, we introduce the F^1^-transform concept; in Section 3, we present our seasonal time series forecasting methods. In Section 4, we show the results of the tests; conclusions and future prospects are contained in Section 5.

## 2. F^1^-Transform

### 2.1. Direct and Inverse Fuzzy Transform

Let [*a*,*b*] be a closed interval of real numbers, and *x*_1_, *x*_2_, …, *x_n_* (*n* ≥ 2) be points of [*a*,*b*], called nodes, such that *x*_1_ = *a* < *x*_2_ < … < *x_n_* = *b*. The family of fuzzy sets *A*_1_, …, *A_n_*: [*a*,*b*] → [0,1], called basic functions [18], is a fuzzy partition of [*a*,*b*] if the following holds:

(1) *A_i_*(*x_i_*) = 1 for every *i* = 1, 2, …, *n*;
(2) *A*_*i*_(*x*) = 0 if *x* is in [*x*_*i*−1_,*x*_*i*+1_] for *i* = 2, …, *n* − 1;
(3) *A_i_*(*x*) is *a* continuous function on [*a*,*b*];
(4) *A_i_*(*x*) strictly increases on [*x*_*i*−1_, *x_i_*] for *i* = 2, …, *n* and strictly decreases on [*x_i_*,*x_i_*_+1_] for *i* = 1,…, *n* − 1;
(5) *A*_1_(*x*) + … + *A_n_*(*x*) = 1 for every *x* in [*a*,*b*].
  The fuzzy sets {*A*_1_(*x*), …, *A_n_*(*x*)} form an h-uniform fuzzy partition of [*a*,*b*] if
(6) *n* ≥ 3 and *x_i_* = *a* + *h*∙(*i* − 1), where *h* = (*b* − *a*)/(*n* − 1) and *i* = 1, 2, …, *n* (that is, the nodes are equidistant);
(7) *A_i_*(*x_i_* − *x*) = *A_i_*(*x_i_* + *x*) for every *x* in [0,*h*] and *i* = 2, …, *n* − 1;
(8) *A*_*i*+1_(*x*) = *A_i_*(*x* − *h*) for every *x* in [*x_i_*, *x*_*i*+1_] and *i* = 1, 2, …, *n* − 1.

Let *f*(*x*) be a function defined in [*a*,*b*]. Here, we are only interested in the discrete case, that is, in functions *f*, assuming determined values in the set *P* of points *p*_1_, ..., *p_m_* of [*a*,*b*]. The set *P* is called sufficiently dense with respect to the fixed partition {*A*_1_, *A*_2_, …, *A_n_*} if, for any index *i* in {1, …, *n*}, there exists at least an index *j* in {1, …, *m*} such that *A_i_*(*p_j_*) > 0

If *P* is sufficiently dense with respect to the fixed fuzzy partition {*A*_1_, *A*_2_, …, *A_n_*}, we can define the n-tuple {*F*_1_, *F*_2_, …, *F_n_*} as the discrete direct F-transform of *f* with respect to the basic functions {*A*_1_, *A*_2_, …, *A_n_*} [18], with the following components:

(1) $$F_{k}=\frac{\sum_{i=1}^{m}f(p_{i})A_{k}(p_{i})}{\sum_{i=1}^{m}A_{k}(p_{i})}$$

for *k* = 1, …, *n*. Similarly, we define the discrete inverse F-transform of *f* with respect to the basic functions {*A*_1_, *A*_2_, …, *A_n_*} by setting

(2) $$f_{n}^{F}(p_{i})=\sum_{k=1}^{n}F_{k}A_{k}(p_{i})$$

The following theorem holds (Reference [18]):

**Theorem** **1.** *Let f(x) be a function assigned on the set of points P = {p_1_, ..., p_m_} of [a,b]. Then, for every ε > 0, there exists an integer n(ε) and a related fuzzy partition {A_1_, A_2_, …, A_n(ε)_} such that for any j = 1, …, m*

(3) $$\left|f(p_{j})-f_{n(\varepsilon )}^{F}(p_{j})\right|<\varepsilon$$

### 2.2. F^1^-Fuzzy Transform

Let {*A*_1_(*x*), …, *A_n_*(*x*)} be an uniform fuzzy partition of [*a*,*b*] and $f(x)\in L_{2}[a,b]$, where $L_{2}[a,b]$ denotes the Hilbert space of square integrable functions on [*a*,*b*]. We consider the linear subspace $L_{2}^{1}[a,b]$ of $L_{2}[a,b]$ with orthogonal basis given by the following polynomials:

(4) $$\begin{array}{l} S_{k}^{0}(x)=1\\ S_{k}^{1}(x)=x-x_{k} \end{array}$$

where the coefficients $c_{k}^{0}$ and $c_{k}^{1}$ are given by

(5) $$c_{k}^{0}=\frac{{\langle f,S_{k}^{0}\rangle }_{k}}{{\langle S_{k}^{0},S_{kl}^{0}\rangle }_{k}}=\frac{\int_{x_{k-1}}^{x_{k+1}}f(x)A_{k}(x)dx}{\int_{x_{k-1}}^{x_{k+1}}A_{k}(x)dx}$$

and

(6) $$c_{k}^{1}=\frac{{\langle f,S_{k}^{1}\rangle }_{k}}{{\langle S_{k}^{1},S_{k}^{1}\rangle }_{k}}=\frac{\int_{x_{k-1}}^{x_{k+1}}f(x)(x-x_{k})A_{k}(x)dx}{\int_{x_{k-1}}^{x_{k+1}}A_{k}(x){\left(x-x_{k}\right)}^{2}dx}$$

The following theorem holds (Reference [23], Theorem 3).

**Theorem** **2.** *Let* $f(x)\in L_{2}^{}(\left[a,b\right])$ *and {A_k_(x) k = 1, ..., n} be a h-uniform fuzzy partition of [a,b]. Moreover, let f and A_1_, A_2_, …, A_n_ be functions four times continuously differentiable on [a,b]. Then, the following approximation holds true:*

(7) $$c_{k}^{1}=f^{\prime }(x_{k})+O(h)\;\;\;k=1,\;\ldots ,\;n$$

*where* $f^{\prime }(x_{k})$ *is the derivative of the function f in the point x_k_.*

From Theorem 2 descends the following corollary (Reference [23], Corollary 1).

**Corollary** **1.** *Let*$f(x)\in L_{2}^{}(\left[a,b\right])$*and {A_k_(x) k = 1, ..., n} be a generalized fuzzy partition of [a,b]. Moreover, let*$f_{}$*and A_k_ be four times continuously differentiable on [a,b]. Then, for each k = 1, …, n, we have the following:*

(8) $$f(x)=F_{k}^{1}(x)+O(h^{2})\;\;\;x_{k-1}\leq x\leq x_{k+1}$$

where

(9) $$F_{k}^{1}(x)=c_{k}^{0}+c_{k}^{1}(x-x_{k})$$

*is the kth component of the F^1^-transform of f with respect to A_k_, k = 1, ..., n*.

*Let {A_k_(x) k = 1, ..., n} be an h-uniform fuzzy partition of [a,b] and (x_1_, f(x_1_)),…, (x_n_, f(x_n_)) be a discrete set of n points of the function f. Equations (2) and (3) can approximate f in the discrete case as*

(10) $$c_{k}^{0}=\frac{\sum_{i=0}^{m}f(x_{i})A_{k}(x_{i})}{\sum_{i=0}^{m}A_{k}(x_{i})}$$

*and*

(11) $$c_{k}^{1}=\frac{\sum_{i=0}^{m}f(x_{i})(x_{i}-x_{k})A_{k}(x_{i})}{\sum_{i=0}^{m}{\left(x_{i}-x_{k}\right)}^{2}A_{k}(x_{i})}$$

*respectively. The discrete approximation of*$c_{k}^{0}$*and*$c_{k}^{1}$*with Equations (10) and (11) are used to calculate the discrete F^1^-transform components in Equation (8) and to approximate the function f(x) in Equation (7). The parameter*$c_{k}^{0}$*is given by the kth component of the discrete direct F-transform (Equation (1))*.

*We define the discrete inverse F^1^-transform of f:*

(12) $$f_{n}^{1}(x)=\frac{\sum_{k=1}^{n}F_{k}^{1}(x)A_{k}(x)}{\sum_{k=1}^{n}A_{k}(x)}$$

The following theorem holds:

**Theorem** **3.** *Let {A_k_(x) k = 1, ..., n} be an h-uniform generalized fuzzy partition of [a,b], and let* $f_{n}^{1}(x)$ *be the inverse F^1^-transform of f given by Equation (12). Moreover, let f, A_1_, A_2_, …, A_n_ be functions four times differentiable on [a,b]. Then, for any x ∊ [a,b], the following holds:*

(13) $$f(x)-f_{n}^{1}(x)=+O(h^{2})$$

**Proof of Theorem** **3.** $$\begin{array}{l} f(x)-f_{n}^{1}(x)=f(x)-\frac{\sum_{k=1}^{n}F_{k}^{1}(x)A_{k}(x)}{\sum_{k=1}^{n}A_{k}(x)}=\frac{f(x)\sum_{k=1}^{n}A_{k}(x)-\sum_{k=1}^{n}F_{k}^{1}(x)A_{k}(x)}{\sum_{k=1}^{n}A_{k}(x)}\\ =\frac{\sum_{k=1}^{n}A_{k}(x)\left(f(x)-F_{k}^{1}(x)\right)}{\sum_{k=1}^{n}A_{k}(x)}=O(h^{2})\;\mathrm{by}\;\mathrm{corollary}\;1. \end{array}$$

By Theorem 3, we can use the inverse F^1^-transform to approximate the function *f* in a point *x* ∊ [*a*,*b*]. □

## 3. The Time Series Seasonal Forecasting F^1^ Fuzzy Transform Method (TSSF1)

Let {(*t*^(1)^, *y*_0_^(1)^), (*t*^(2)^, *y*_0_^(2)^) ... (*t*^(m)^, *y*_0_^(m)^)} be a time series formed by a set of M measures of a parameter *y*_0_ at different times; we suppose that this time series shows seasonality.

As in TFSS, we apply a polynomial fitting to approximate the trend of the time series; then, we partition the time series in s seasonal subsets. 

To approximate the seasonality, we calculated the direct F^1^-transform of each subset and approximate the seasonal functionality with the inverse F^1^-transform.

After assessing the functional trend of the phenomenon in time, we subtract the trend from the data, obtaining the de-treated dataset:

(14) $$y^{\left(i\right)}=y_{0}^{\left(i\right)}-trend(t^{\left(i\right)})\;\;\;i=1,\;\ldots ,\;m$$

It is partitioned in *S* subsets, with *S* as the seasonal period. Each subset represents the seasonal fluctuations with respect to the trend.

Let {(*t*^(1)^ , *y*^(1)^), (*t*^(2)^ , *y*^(2)^) ... (*t*^(m^_s_^)^ , *y*^(m^_s_^)^)}, *s* = 1, 2, …, *S* be the *s*th subset given by m_s_ couples of de-treated data where *t*^(1)^, *t*^(2)^, … *t*^(ms)^ are defined in a domain $\left[t_{s}^{-},t_{s}^{+}\right]$. Let {*A*_1_, *A*_2_, …, *A_ns_*} be an h-uniform generalized fuzzy partition sufficiently dense with respect to this subset, where *A*_1_, *A*_2_, …, *A_ns_* are four times differentiable in the domain $\left[t_{s}^{-},t_{s}^{+}\right]$.

We calculate the direct F^1^-transform components (Equation (9)), $F_{k}^{1}(t)=c_{k}^{0}+c_{k}^{1}(t-t_{k})$, where

(15) $$c_{k}^{0}=\frac{\sum_{i=0}^{m_{s}}y^{\left(i\right)}A_{k}(t^{\left(i\right)})}{\sum_{i=0}^{m_{s}}A_{k}(t^{\left(i\right)})}\;\;\;k=1,\;\ldots ,\;n_{s}$$

where

(16) $$c_{k}^{1}=\frac{\sum_{i=0}^{m_{s}}f(t^{\left(i\right)})(t^{\left(i\right)}-t_{k})A_{k}(t^{\left(i\right)})}{\sum_{i=0}^{m_{s}}{\left(t^{\left(i\right)}-t_{k}\right)}^{2}A_{k}(t^{\left(i\right)})}\;\;\;k=1,\;\ldots ,\;n_{s}$$

We approximate the seasonal fluctuation at time *t* with the following inverse F^1^-transform:

(17) $$f_{n_{s}}^{1}(t)=\frac{\sum_{k=1}^{n_{s}}F_{k}^{1}(t)A_{k}(t)}{\sum_{k=1}^{n_{s}}A_{k}(t)}$$

To forecast the value of the parameter *y*_0_ at time *t* in the *h*th season, we apply the following formula:

(18) $${\tilde{y}}_{0}(t)=f_{n_{s}}^{1}(t)+trend(t)$$

where ${\tilde{y}}_{0}(t)$ is the approximation of the parameter *y*_0_ at time *t*, $f_{n_{s}}^{1}(t)$ is the *s*th seasonal fluctuation at time *t*, and *trend*(*t*) is the trend of *y*_0_ at time *t*.

For creating the h-uniform fuzzy partition of the *s*th subset, we take the following basic functions:

(19) $$\begin{array}{l} A_{1}(t)=\left\{ \begin{array}{ll} 0.5\cdot (1+\cos\frac{\pi }{h_{s}}(t-t_{1})) & \mathrm{if}\;t\in {\;[t}_{1}{,t}_{2}]\\ 0 & \mathrm{otherwise} \end{array}\right.\\ A_{k}(t)=\left\{ \begin{array}{ll} 0.5\cdot (1+\cos\frac{\pi }{h_{s}}(t-t_{k})) & \mathrm{if}\;t\in {\;[t}_{k-1}{,t}_{k+1}]\;\\ 0 & \mathrm{otherwise} \end{array}\right.\\ A_{n_{s}}(t)=\left\{ \begin{array}{ll} 0.5\cdot (1+\cos\frac{\pi }{h_{s}}(t-t_{n_{s}})) & \mathrm{if}\;t\;\in \;[t_{n_{s}-1},t_{n_{s}}]\\ 0 & \mathrm{otherwise} \end{array}\right. \end{array}$$

where *t*_1_ = $t_{s}^{-}$, *t*_2_, … *t_ns_* = $t_{s}^{+}$ are the nodes, $h_{s}=\frac{t_{s}^{+}-t_{s}^{-}}{n_{s}-1}$, and $t_{k}=t_{s}^{-}+h_{s}(k-1)$ *k* = 1, …, *n_s_*.

To obtain the optimal number of nodes *n_s_*, we implement the process applied in Reference [17]: the value of *n_s_* is initially set to 3. Then, we calculate the direct F^1^-transform components via Equations (15) and (16) and the Mean Absolute Deviation Mean (MAD-MEAN) index, given by

(20) $$MAD-MEAN=\frac{\sum_{i=1}^{m_{s}}\left|f_{n_{s}}^{1}(t^{\left(i\right)})-y^{\left(i\right)}\right|}{\sum_{i=1}^{m_{s}}y^{\left(i\right)}}$$

where the value $f_{n_{z}}^{1}(t^{\left(i\right)})$ *i* = 1, 2, …, *m_s_* is calculated by Equation (17). The MAD-MEAN index represents a good accuracy metric in time series analyses, as proved in Reference [24].

If the MAD-MEAN index is greater than a specified threshold, the algorithm stops and Equation (18) is used to assess the value of *y*_0_ at time *t*; otherwise, the process is iterated by creating an h-uniform fuzzy partition, where *n_s_* = *n_s_* + 1. At any iteration, if the subset is not sufficiently dense with respect to the fuzzy partition, the algorithm stops; else, the values of $c_{k}^{0}$ and $c_{k}^{1}$, *k* = 1, 2, …, *n_s_* by Equations (15) and (16) are calculated.

Table 1 shows the algorithm in pseudocode. The output of the algorithm are the polynomial coefficients to be used to obtain the trend at time *t* and the F^1^-transform components $c_{k}^{0}$ and $c_{k}^{1}$, so to calculate the assess of the value ${\tilde{y}}_{0}(t)$ at time *t* via Equation (18).

**Figure 1:**
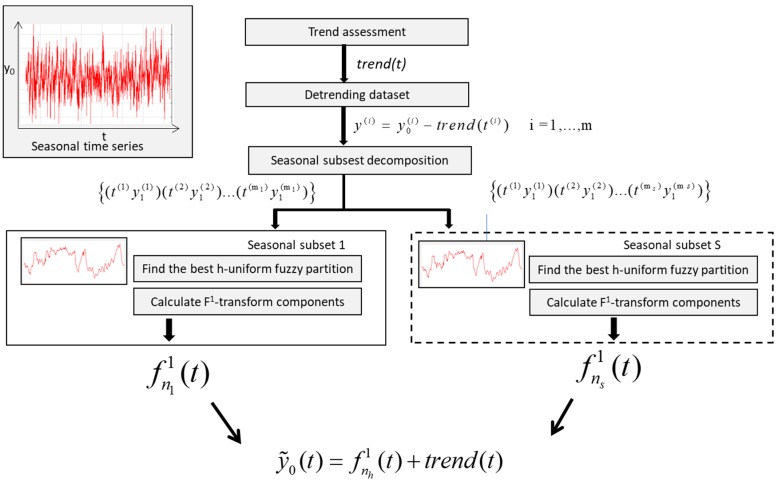
Schema of the TSSF1 algorithm.

Figure 1 is a schematized TSSF1 algorithm. 

## 4. Experimental Results

We test the TSSF1 algorithm on a dataset of daily weather data collected from weather stations. The dataset is composed by daily weather data collected from the weather stations managed by the Italian Air Force located in the Campania Region: they are the weather stations of Capo Palinuro, Capri, Grazzanise, Napoli Capodichino, Salerno Pontecagnano, and Trevico. 

Our aim is to analyze the seasonality of the Heat Index (HI) [25], an index function of the maximum daily air temperature and of the daily relative humidity. HI index measures the physiological discomfort caused by the presence of high temperatures and high humidity levels.

The HI takes into account several factors, such as vapor pressure, actual wind speed, sample size, internal body temperature, and sweating rate, represented by numerical coefficients. The calculation of HI is based on the following formula obtained by multiple regression analysis carried out in Reference [26] (NWS-NOAA, 2):

(21) $$HI=c_{1}+c_{2}T+c_{3}RH+c_{4}T\cdot RH+c_{5}T^{2}-c_{6}RH^{2}+c_{7}T^{2}\cdot RH+c_{8}T\cdot RH^{2}{}^{}+c_{9}T^{2}\cdot RH^{2}$$

with *T* = air temperature and *RH* = relative humidity (%). The values of the coefficients *c*_1_, ..., *c*_9_ are shown in Appendix A.

This formula applies only in the case of temperatures above 27 °C and relative humidities above 40%, conditions often verified during the summer. For temperatures below 25 °C, with low humidity (<30%), it can be assumed that the heat index coincides with the actual temperature, without significant effects due to humidity. 

The table in Appendix A shows the classification of the heat wave health hazard levels based on HI values carried out by the United States National Weather Service-National Oceanic Atmospheric Administration (NWS-NOAA, 2).

The training datasets are given by HI values measured in degrees Celsius and calculated by the daily max temperature and the relative humidity recorded in the months July and August from 1 July 2003 to 31 August 2017, comprising a period of 918 days. The season is given by the number of weeks, so we partition each dataset in *k* = 9 subsets.

Following the TSSF algorithm, we calculate the trend fitting the data with a polynomial of 9th degree $y=\sum_{i=0}^{9}a_{i}\cdot t^{i}$; then, a threshold value of 5 for the MAD-MEAN index is set.

**Figure 2:**
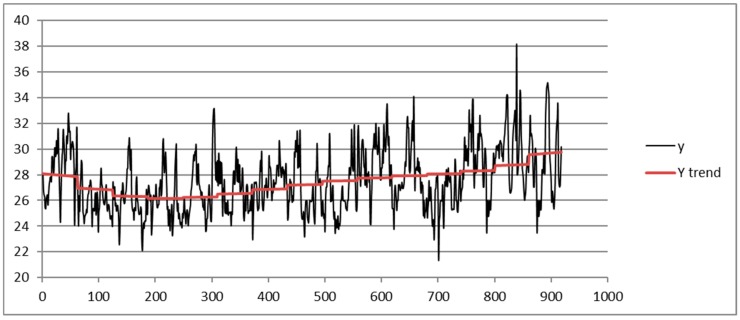
Trend of the heat index (HI) in the months of July and August (from 1 July 2003 to 16 August 2017) obtained from the Napoli Capodichino station dataset by using a ninth-degree polynomial fitting.

Figure 2 shows the trend obtained from the dataset of the station of Capodichino. The day is represented on the abscissa using the corresponding progressive identifier. 

We compare the results obtained via SARIMA, ADANN, TSSF, and TSSF1. We use the Forecast Pro tool [27] to apply the SARIMA algorithm. The ADANN method is applied by implementing the ADANN algorithm in References [17,28,29]; based on the experimental tests we have carried out, we apply a GA algorithm with a stopping criterion of 200 generations to search the optimal number of the input and hidden layer nodes. The TSSF method is applied implementing the TSSF algorithm in Reference [22]. 

**Figure 3:**
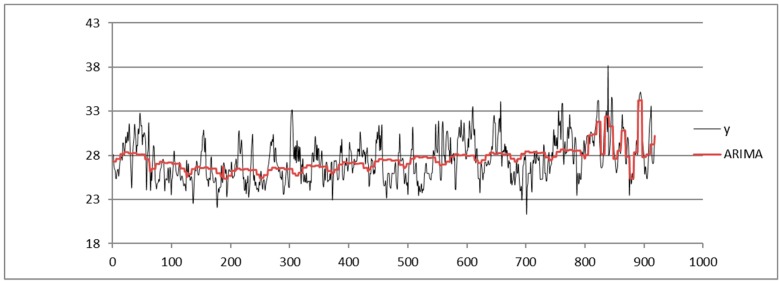
Plot of HI index time series from the Napoli Capodichino station dataset obtained by using the Seasonal Autoregressive Integrated Moving Average (ARIMA) algorithm.

**Figure 4:**
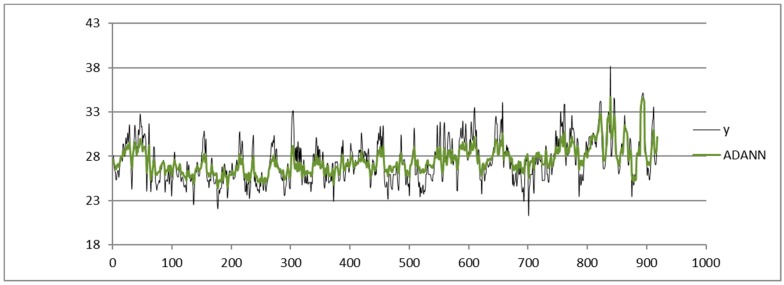
Plot of HI index time series from the Napoli Capodichino station dataset obtained by using the Automatic Design of Artificial Neural Networks (ADANN) algorithm.

**Figure 5:**
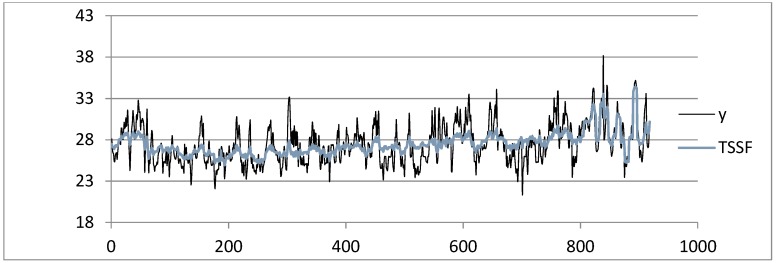
Plot of HI index time series from the Napoli Capodichino station dataset obtained by using the TSSF algorithm.

**Figure 6:**
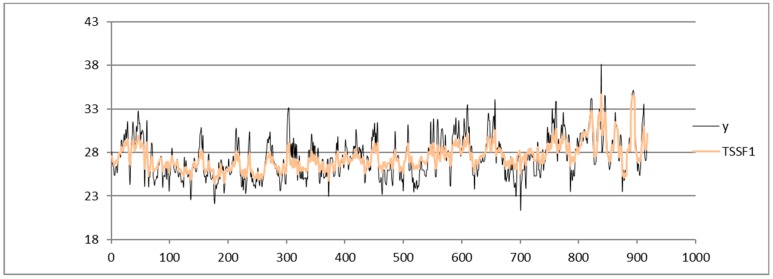
Plot of HI index time series from the Napoli Capodichino station dataset obtained by using the TSSF1 algorithm.

Shown below is the HI index time series from the dataset of the Napoli Capodichino station obtained by applying the SARIMA (Figure 3), ADANN (Figure 4), TSSF (Figure 5), and TSSF1 (Figure 6) algorithms.

We compare the results obtained via SARIMA (Figure 3).

To measure the performances of the algorithms in addition to the MAD-MEAN index, we calculate also the well-known time series accuracy indexes: Root Mean Square Error (RMSE), Mean Absolute Percentage Error (MAPE), and Mean Absolute Deviation (MAD).

In Table 2, the measures of the four accuracy indexes obtained from all the datasets of the weather stations are shown. For each dataset, the ARIMA, ADANN, TSSF, and TSSF1 algorithms are applied as well.

The results in Table 2 show that, for all the datasets, the performance of the TSSF1 algorithm are better than that of the Spatial ARIMA and TSSF algorithms and comparable with that of the ADANN algorithm. In fact, both the measured values of the MAD-MEAN index and those of the RMSE, MAD, and MAPE indices obtained by using the TSSF1 method are very similar to the values obtained using the ADANN method; on the other hand, the ADANN method has a higher computational complexity with respect to the TSSF1 algorithm due to the use of the GA algorithm necessary for determine the optimal number of nodes of the input layer and the hidden layer.

In order to measure the forecasting performances of the results for any weather station, we create a test dataset given HI values related to the period 1 July 2018–31 August 2018; then, we calculate the RMSE of the forecasted values obtained by using the SARIMA, ADANN, TSSF, and TSSF1 algorithms. In Table 3, we show the RMSE measured in the 9 methods for each parameter.

As well as the results in Table 2, the results in Table 3 show that the forecasting performances of the TSSF1 algorithm are comparable with that of the ADANN algorithm and better than that of the SARIMA and TSSF algorithms. This trend is confirmed for all six datasets used in this comparison test. 

## 5. Conclusions

We propose a novel seasonal time series forecasting algorithm based on the direct and inverse F^1^-transform. The aim of this research is to improve the performance of the TSSF algorithm, a seasonal time series forecasting method based on direct and inverse F-transform. As in TSSF, we apply a polynomial fitting to extract the trend and partition the training dataset in S subsets, where S is the number of seasons. For each subset, the direct F^1^-transform components are calculated and the inverse F^1^-transform is used to predict the value of an assigned output as well.

We test our algorithm on datasets of the daily heat index in the months of July and August calculated by using the daily max temperature and humidity values measured from the six Italian weather stations of Capo Palinuro, Capri, Grazzanise, Napoli Capodichino, Salerno Pontecagnano, and Trevico starting from 1 July 2003 and up to 31 August 2017. We compare the accuracy and the forecasting performances of our method with the ones obtained by using the Seasonal ARIMA ADANN and TSSF methods; the results show that the proposed method has better performances than those obtained using Seasonal-ARIMA and TSSF and performances comparable with those obtained by using the ADANN algorithm, with the advantage of being more efficient than ADANN in terms of computational complexity; in fact, compared to the TSSF1 algorithm, which has a quadratic dependence on the size of the dataset, ADANN has longer execution times, since in ADANN, two hundred generations are needed to obtain the optimal number of input and hidden layer nodes.

In the future, we intend to optimize the performance of the TFSS1 algorithm, parallelizing the calculation processes of the direct F-transform components on each seasonal subset and implementing an efficient algorithm for optimizing the MAD-MEAN index threshold.

## Figures and Tables

**Table 1:** Pseudocode of the Time Series Seasonal Forecasting F1 Fuzzy Transform (TSSF1) algorithm.

(1) Calculate the trend using a polynomial fitting (2) Store the polynomial coefficients (3) Subtract to the data the trend value obtaining a new dataset (4) Partition the dataset into subsets; each data subset contains the measured data in a season. (5) For each seasonal subset (6) *n*: =3 (7) stop: = FALSE (8) WHILE (stop = FALSE) (9) Set the h-uniform fuzzy partition (19) (10) IF the subset is sufficiently dense with respect to the fuzzy partition (11) Calculate the direct F^1^-transform components by (15) and (16) (12) Store *c_k_*^0^ and *c_k_*^1^ *k* = 1, 2, …, *n_s_* (13) Calculate the MAD-MEAN index (20) (14) *n*: =*n* + 1 (15) IF MAD-MEAN > Threshold THEN (16) stop: =TRUE (17) END IF (18) ELSE (19) stop: =TRUE (20) END IF (21) END WHILE (22) NEXT

**Table 2:** Accuracy measures for HI index time series from all the weather station datasets obtained by using ARIMA, ADANN, TSSF, and TSSF1.

Station	Forecasting Method	RMSE	MAPE	MAD	MAD-MEAN
Capo Palinuro	ARIMA	1.65	5.56	1.54	4.95
	ADANN	1.43	5.22	1.24	4.38
	TSSF	1.49	5.37	1.34	4.56
	TSSF1	1.43	5.22	1.26	4.37
Capri	ARIMA	1.75	5.63	1.64	5.00
	ADANN	1.53	5.28	1.36	4.41
	TSSF	1.59	5.43	1.47	4.60
	TSSF1	1.52	5.30	1.37	4.41
Grazzanise	ARIMA	1.72	5.59	1.61	4.96
	ADANN	1.50	5.30	1.38	4.49
	TSSF	1.61	5.47	1.45	4.58
	TSSF1	1.53	5.29	1.36	4.45
Napoli Capodichino	ARIMA	1.68	5.48	1.41	4.93
	ADANN	1.46	5.14	1.17	4.35
	TSSF	1.52	5.29	1.26	4.54
	TSSF1	1.45	5.16	1.18	4.35
Salerno	ARIMA	1.74	5.63	1.61	4.98
	ADANN	1.52	5.34	1.38	4.51
	TSSF	1.63	5.51	1.45	4.60
	TSSF1	1.55	5.33	1.36	4.47
Pontecagnano	ARIMA	1.62	5.43	1.35	4.87
	ADANN	1.41	5.07	1.13	4.30
	TSSF	1.51	5.16	1.20	4.45
	TSSF1	1.39	5.06	1.13	4.29
Trevico	ARIMA	1.76	5.67	1.62	5.01
	ADANN	1.56	5.36	1.39	4.50
	TSSF	1.64	5.54	1.47	4.65
	TSSF1	1.55	5.36	1.38	4.51

**Table 3:** RMSE of the test dataset for the HI index time series from all the weather station datasets obtained by using ARIMA, ADANN, TSSF, and TSSF1.

Station	RMSE			
	**ARIMA**	**ADANN**	**TSSF**	**TSSF1**
Capo Palinuro	1.28	1.01	1.19	0.99
Capri	1.33	1.02	1.22	1.02
Grazzanise	1.35	1.04	1.24	1.05
Napoli Capodichino	1.35	1.04	1.22	1.03
Salerno	1.36	1.05	1.24	1.05
Pontecagnano	1.32	1.03	1.20	1.04

## Appendix A

**Table A1:** Parameters used to calculate the heat index, setting the unit measure of the temperature in °C or °F (Reference [19]).

Parameter	°C	°F
*c*1	−8.78469475556	−42.379
*c*2	1.61139411	2.04901523
*c*3	2.33854883889	10.14333127
*c*4	−0.14611605	−0.22475541
*c*5	−0.012308094	−0.00683783
*c*6	−0.0164248277778	−0.05481717
*c*7	0.002211732	0.00122874
*c*8	0.00072546	0.00085282
*c*9	−0.000003582	−0.00000199

**Table A2:** Classification of the heat wave health hazard levels based on HI values (NWS-NOAA, 2).

Alert Level	Heat Index	Possible Heat Disturbances for Vulnerable People
Caution	80 °F (27 °C) ≤ HI < 89 °F (32 °C)	Possible tiredness following prolonged exposure to the sun and/or physical activity
Extreme caution	90 °F (32 °C) ≤ HI < 104 °F (40 °C)	Possible sunstroke, heat cramps with prolonged exposure, and/or physical activity
Danger	105 °F (41 °C) ≤ HI < 129 °F (54 °C)	Probably sunstroke, heat cramps, or heat exhaustion; possible heat stroke with prolonged exposure to the sun and/or physical activity
High danger	HI ≥ 130 °F (54 °C)	High probability of heat stroke or sunstroke caused by continuous exposure to the sun
